# Development of an insilico model of eccrine sweat using molecular modelling techniques

**DOI:** 10.1038/s41598-022-24440-x

**Published:** 2022-11-24

**Authors:** Parijat Deshpande, Bharath Ravikumar, Siddharth Tallur, Debjani Paul, Beena Rai

**Affiliations:** 1 TCS Research, Tata Research Development and Design Centre (TRDDC), Pune, 411013 India; 2 Centre for Research in Nanotechnology and Science (CRNTS), IIT Bombay, Mumbai, 400076 India; 3 Department of Electrical Engineering, IIT Bombay, Mumbai, 400076 India; 4 Department of Biosciences and Bioengineering, IIT Bombay, Mumbai, 400076 India

**Keywords:** Lab-on-a-chip, Computational models

## Abstract

Eccrine sweat is an ideal surrogate diagnostic biofluid for physiological and metabolic biomarkers for wearable biosensor design. Its periodic and non-invasive availability for candidate analytes such as glucose and cortisol along with limited correlation with blood plasma is of significant research interest. An insilico model of eccrine sweat can assist in the development of such wearable biosensors. In this regard, molecular modelling can be employed to observe the most fundamental interactions. Here, we determine a suitable molecular model for building eccrine sweat. The basic components of sweat are water and sodium chloride, in which glucose and other analytes are present in trace quantities. Given the wide range of water models available in the molecular dynamics space, in this study, we first validate the water models. We use three compounds to represent the base to build bulk sweat fluid and validate the force fields. We compare the self-diffusivity of water, glucose, sodium, and chloride ions as well as bulk viscosity values and present the results which are > 90% accurate as compared with the available literature. This validated insilico eccrine sweat model can serve as an aid to expedite the development de novo biosensors by addition of other analytes of interest e.g. cortisol, uric acid etc., simulate various temperatures and salt concentrations, expand search space for screening candidate target receptors by their binding affinity and assess the interference between competing species via simulations.

## Introduction

Eccrine sweat has two distinct advantages over other candidate biofluids owing to its non-invasive, periodic availability and limited correlation with blood serum. This has motivated researchers to consider it as a surrogate diagnostic biofluid since the existence of amino acid serine was detected in 1910^1^. Subsequently, a detailed study of eccrine sweat was conducted by Ray and McSwiney for its composition^2^. Research in this direction was further fuelled by the findings of glucose and lactic acid in sweat by Silvers in 1928^3^. Other constituents such as ammonia, glucose, and chloride as candidate biomarkers were isolated and compared with the levels from patients by Ray and Steck^3^. Similar studies were largely targeted towards differentiating sweat composition amongst healthy subjects and patients which demanded elaborate laboratory experiments in the early twentieth century. Further detailed analysis of the composition of sweat was conducted and established by Robinson and Robinson^4^ which is considered in this paper for model development. Significant interest in other biofluids such as saliva^5,6^ and blood^7^ has motivated the development of insilico models for these fluids using molecular modelling techniques.

However, sweat offers a clear advantage over other biofluids for non-invasive and periodic monitoring of target analytes such as glucose and cortisol. Models for blood and saliva though with limited utility in terms of predicting blood viscosity and the components and properties of saliva have demonstrated their benefits. The detailed physiological mechanisms of determining eccrine sweat have been documented by Baker and Wolfe^8^ in 2020, where they present that sweat composition is not only influenced by extracellular solute concentrations, but also mechanisms of secretion and/or reabsorption, sweat flow rate, by-products of sweat gland metabolism, skin surface contamination, and sebum secretions, among other factors related to methodology. Human eccrine sweat is a biomarker-rich fluid with limited correlations with blood serum. The possibility of detecting these analytes via non-invasive methods using readily available sweat has been abundantly motivated. The advent of nano-biosensing combined with various electrochemistry methods and AI to interpret the results has made it possible to realize such sensors. On-going research on such point-of-care sensors is targeted towards improving their sensitivity and selectivity by testing the various bio-receptor combinations of target molecules and target receptors, ensuring reproducibility, and other allied research such as microfluidics and flexible electronics.

Insilico models for sweat are not present to the best of our knowledge and this work attempts to develop a building block in the direction of insilico model of eccrine sweat. The composition of sweat^9,10^ is well studied and the mechanism of natural sweating and iontophoresis^11^ is available in the literature. Microfluidic models have been explored for eccrine sweat which is imperative to the development of sensor patches and wearables to ensure high-throughput and continuous measurements for various analytes^12^. Developing this insilico model not only aids experimentation but also gives insight into the mechanism and processes involved in the operation of the sensor. Furthermore, the general challenges with respect to sensitivity and selectivity can be studied via simulations using the proposed insilico sweat model. Similar studies have been conducted on other body fluids such as composition and properties of saliva^13^ and bulk properties of blood^14^ such as viscosity using molecular dynamics (MD). Such an insilico model will aid in development of wearable biosensors by simulating various temperature and salt concentration conditions, screen candidate receptors and substrates via their binding energy levels, study competing species interferences etc. Encouraged by these factors we propose to build a molecular model for sweat and validate with existing literature data. There is no known available insilico sweat model for simulating such studies for detecting primary constituent analytes of eccrine sweat such as metabolites, biomolecules, and other analytes. Our model will facilitate and expedite the screening of candidate substrates, functionalizing as per the target molecule of interest and therefore improve selectivity. Studies with competing or interfering species^15^ too can be carried out by using such an insilico model of sweat. This motivates a clear case for developing an eccrine sweat model for simulating insilico experiments.

Since eccrine sweat is a dilute salt solution with biomarkers, in this work, we restrict ourselves to the comparison of the molecular model consisting of glucose in a salt solution of appropriate concentration. Literature data for glucose in salt solutions at various temperatures and concentrations is readily available to compare and can serve as a benchmark in order to build a complete sweat model consisting of all the constituents. This model is developed using Large-scale Atomic/Molecular Massively Parallel Simulator (LAMMPS) tool^16^ and presented along with validation results for diffusivity and viscosity. Additionally, the effect of variation in temperature and concentration of electrolytes is presented as a basis for the development of de novo biosensors. The purpose of modelling is to identify key experimental parameters such as transport properties to serve as an aid in the pursuit of the development of de novo biosensors. These parameters determine the response or output of the sensor and provide a way to explore the relation between key experimental parameters, the concentration of the analytes and sensor response. The computation of viscosity of the solution and diffusivity of analytes such as glucose via modelling validate the efficacy of the insilico model. The values of diffusivity of glucose are available in literature for various concentrations of salt water and various temperature conditions. Comparing these values with our model output will validate the model for further usage. Thus, validated insilico eccrine sweat model can then provide insight into the mechanism and processes involved in the operation and therefore aid development of wearable biosensors by simulating various temperature and salt concentrations, screening candidate receptors and substrates and studying the competing-species interferences.

## Theory and method

### Development of a Matlab^®^ based LAMMPS script generator

**Figure 1:**
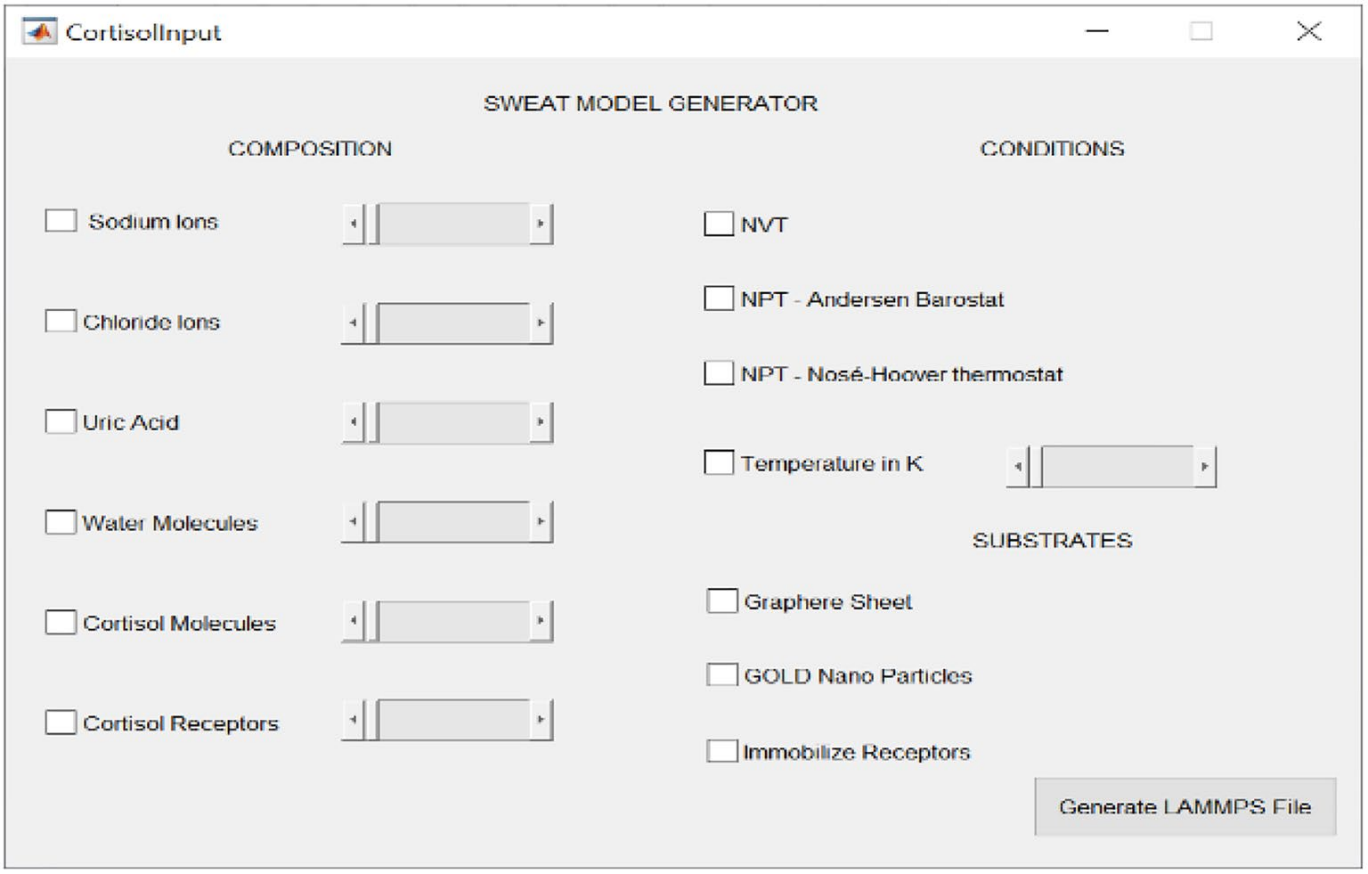
LAMMPS script generator.

We present a Matlab^®^ based tool complete with GUI as depicted in Fig. 1 which allows users to Select constituents, Input conditions (NPT/NVT), and Vary concentrations for the insilico Sweat Model and generate a LAMMPS script. Users can subsequently automate this program for varying conditions, and concentrations e.g., the concentration of sodium ions to simulate dehydration and simulate the diurnal circadian cycle. Simulations with candidate substrates and receptors can be conducted to expedite the development of biosensors with larger search space and faster results as compared to experimental analysis.

### Development of an insilico eccrine sweat model

**Figure 2:**
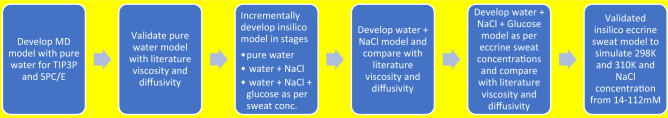
Flow chart of development of insilico eccrine sweat model.

The first step in developing a sweat model involves ensuring the appropriate composition of eccrine sweat. Most of the sweat of our body is produced via eccrine glands as compared to apocrine glands^9^. The eccrine sweat glands are mainly located on the palms, soles, forehead, and armpits and cover the rest of the body^17^. Sweat secreted by these glands is primarily water and the remaining constituents, specifically significant for wearable sensing are listed in Table 1. These concentrations are further translated into the number of molecules according to their molarity. Sweat is considered as a dilute water solution with NaCl as the primary solute and other analytes of interest in much smaller ratios^8^. Na^+^ or Cl^−^ can be directly measured using ion-selective electrodes^6,7^ or electrical conductivity of the sweat can be measured, since Na^+^ and Cl^−^ are the abundant ions in sweat. Small molecules (< 1000 Da) such as glucose (180.156 g/mol or Da) are present in trace amounts. It is therefore imperative that the water model selected for this work is thoroughly validated to ensure the subsequent complex models developed will retain their accuracy. A sequential approach is considered to develop the proposed sweat model progressing from a pure water system to a solution as depicted in Fig. 2. The individual biomolecules are selected from protein data bank^18^ and added to the water molecules. Since glucose diffusivity values are readily available in literature both by simulations as well as experiments, it is selected as the candidate analyte. Glucose is therefore added to the salt solution as a surrogate for several biomolecules and to validate the presented model with literature data. Thus, the development will have to begin with a complete molecular model of human sweat with the same composition with careful matching of the primary constituents in their appropriate concentrations as listed in Table 2 and rendering presented in supplementary information Figure S6.

**Table 1:** Typical composition of eccrine sweat as reported in literature^4,9,10^.

Constituents	Concentration/Molarity
Cortisol	0.022–0.386 µM
Glucose	10–200 µM
Uric Acid	2–10 mM
Na^+^	10–100 mM
Cl^-^	10–100 mM
K^+^	1–18.5 mM
Ca^++^	0.41–12.4 mM
NH4	0.1–1 mM
Ethanol	2.5–22.5 mM
Ascorbic Acid	10–50 µM

**Table 2:** Molecular model constituents and their concentration for developing insilico sweat model.

Constituents	Concentration of insilico sweat model
Water molecules	Solvent
Glucose	200 µM
Na^+^	14–112 mM
Cl^−^	14–112 mM

### Water models and force fields

As water is the major component of sweat, the suitability of candidate water models effectively determines the usability of the widely used models of water in developing the molecular model of sweat. Thus, to develop a full atomistic sweat model, various candidate water models have to be validated against the experimental values and established simulation results. Water models TIP3P and SPC/E are implemented and their transport properties such as viscosity and self-diffusivity are validated. Both these empirical water models used in this simulation are similar and have three interaction sites, but with differences in their pair potentials composed of Lennard–Jones (LJ) and Coulombic terms resulting in differences in the calculated self-diffusion coefficients of water^19,20^. The SPC/E model assumes a water molecule as a rigid molecule as a rigid molecule with an intramolecular distance of 0.1 nm between oxygen and hydrogen interaction sites (O–H distance) and with an angle of 109.47° between the O–H bonds. The intermolecular site–site interactions are defined in terms of the distances between the sites. Although these sites are commonly interpreted in terms of oxygen and hydrogen atoms, they are merely sites for atom–atom and Coulomb interactions. There are partial charges assigned to the sites to mimic an effective charge distribution of a water molecule in liquid water^20^. The charge on the oxygen site is -0.8476e and the charge on the hydrogen site is 0.4238e. The SPC/E model assumes an ideal tetrahedral shape (HOH angle of 109.47°) instead of the observed angle of 104.5^19^. The TIP3P model^19^ is similarly specified as a 3-site rigid water molecule with charges and Lennard–Jones parameters assigned to each of the three atoms. The charge on the oxygen site is -0.830e and the charge on the hydrogen site is 0.415e. The model assumes an HOH angle of 104.52°. The SHAKE algorithm was used to keep the bonds of water molecules rigid^21,22^.

The force field selected was CHARMM36m due to its suitability for biomolecules^23^. The present work utilizes the CHARMM36m^24^ force field equations. CHARMM36m is all-atom additive protein force field with validation based on comparison to NMR data^25^. The implementation of this force field was achieved by CHARMM–GUI^26^. The simulation input files consisting of the TIP3P model were manually altered to include the SPC/E parameters for improved results. Development of a molecular dynamics model of eccrine sweat demands both validation of composition and validation of transport coefficients such as diffusivity values and bulk properties such as viscosity and density along with the data in the literature both empirical and experimental with the small molecules such as glucose and sodium and chloride ions.

### LAMMPS simulation

The insilico sweat model was developed by using LAMMPS—molecular modelling tool with the above composition by developing the data file using CHARM-GUI^24^. All simulations were carried out using 11,465 water molecules in a cubic simulation cell of volume 70 × 70 × 70 Å^3^ with periodic boundary conditions. The composition of the various analytes is collected from the RCSB protein database^18^ and imported into LAMMPS via CHARM-GUI Solution builder. The choice of timestep for a biological system is based on the vibrational frequency of the individual molecules which is of the order of 10^15^ Hz i.e., T = 1/f = 1 fs^27^. A cut-off of 10 Å is used to compute the van der Waals interactions via the LJ potential term in the force-field. The long-range coulombic interactions are computed using particle–particle particle-mesh solver (PPPM) methodology by solving in K-space.

All the simulated systems follow an identical simulation procedure. Initially, the system energy is minimized from its initial configuration using the conjugate gradient method. This allows the placement of various atoms and molecules in the control volume avoiding overlap and respecting the minimum distance criterion^28^. Post minimization, the system is equilibrated for 5 ns under NPT conditions to achieve the density at 1 atm. Subsequently, the simulations are carried out at NVT for 5 ns. Here, N, V, T and P denote the number of atoms, volume, temperature, and pressure respectively. Subsequently, these simulations are made to run for another 5 ns in the production run at NPT to ensure they produce representative macroscopic values based on the statistical mechanic postulates of ergodicity i.e., the time average = ensemble average. For each composition, and temperature condition, the production phase is run for 5 ns. Atomic coordinates stored during the production phase are utilized to compute time-averaged results of static and dynamic properties such as diffusivity and viscosity at various temperatures. Representative plots are shown in Figure S2 in supplementary information. A sample simulation production run of 30 ns as presented in Figure S3 in supplementary information, was conducted to ensure results of the 5 ns runs are in close agreement and equilibrium is achieved.

In the following section, we initially compare the properties of pure water system with the literature to identify the suitable water model to develop the insilico sweat model. Subsequently, the diffusivities of the components in the simulation system of aqueous NaCl are compared. Following that, the diffusivities of components in aqueous glucose system are computed. Finally, the values of diffusivity of components in the biological solution (insilico sweat model) at different temperatures in the presence of different concentrations of NaCl are computed and compared to that of experimental values in literature.

### Ethics statement

The entire data presented in this publication is simulated data and no human/animal tests were conducted.

## Results and discussion

### Validation of water model with pure water system

#### Comparison of viscosity of water

Simulations of our models (both TIP3P and SPC/E) at the same temperature 298 K conditions are presented along with other literature studies in Table 3 to serve as a first stage validation^19^.

**Table 3:** Dynamic Viscosity of pure water model compared with TIP3P and SPC/E water models and experimental values.

Dynamic Viscosity at 298 K	TIP3P Model [mPa s]	SPC/E Model [mPa s]
Insilico Model (water)	0.325	0.710
Simulation Literature Values ^19^	0.321	0.729
Experimental Values^29^	0.890	

Table 3 shows the values of viscosity for the two different water models. The SPC/E water model shows a viscosity value of 0.729 mPa s. The TIP3P water model shows a viscosity value of 0.321 mPa s. The experimental values for dynamic viscosity of water are 0.89 mPa s^22,29^ indicating that SPC/E model appears to be better suited than the TIP3P model. Both these simulations are carried out for a total production run of approx. 5 ns with each timestep of 1 fs with the implementation of appropriate water models namely, TIP3P and SPC/E. Since these simulations are carried out with a sufficiently large number of water molecules i.e., 11,465 and for 5 × 10^6^ steps i.e., 5 ns, the results are depicted as mean values and in close agreement with the literature. These models are empirical in nature and the SPC/E parameters offer a better performance, the same is supported in the literature and scaling is suggested for using the SPC/E model^19^. The dynamic viscosity of the solution offers an opportunity to validate the bulk properties which are readily available both experimentally and via simulation in the literature. We compute the bulk viscosity based on the Green–Kubo formula which relates the ensemble average of the autocorrelation of the stress/pressure tensor to *η*. *η* is a measure of the propensity of a fluid to transmit momentum in a direction perpendicular to the direction of velocity or momentum flow.

$$\eta = \frac{V}{kB T }\mathop \smallint \limits_{0}^{\infty } dt\langle Pxy\left( 0 \right)|Pxy\left( t \right)\rangle$$

where V is a volume of the particle system, T is a temperature, k_B_ is the Boltzmann constant, $\langle{....}\rangle$ is averaging over the ensemble, Pxy is the off-diagonal element of the stress tensor^30^. Subsequently, these water models TIP3P and SPC/E have been implemented and their effects assessed on the transport properties such as diffusivity and bulk properties such as density and viscosity to ascertain their utility.

#### Comparison of water self-diffusivity

The diffusivity of a particle indicates the pace at which the particle is transported and computed from the mean square displacement (MSD) of the particles. In Einstein’s theory, the diffusion coefficient can be calculated by using the formula:

$$D = \frac{1}{6t}\left\langle {\left[ {\mathop \sum \limits_{i = 1}^{N} ri\left( t \right) - ri\left( 0 \right)} \right]^{2} } \right\rangle$$

where N is the number of particles, 0 is the reference time,$ri$ is a radius vector of a particle^30^. The diffusivity of given ions and molecules is computed from LAMMPS and plotted as diffusivity vs time and compared with literature values. These computations are carried out via LAMMPS in-built commands and corresponding diffusivity values are available as output. These values are compared with the computed time average values of MSD for each molecule from its trajectory stored (at regular intervals of 100 fs) in a production phase run of 5 ns and 30 ns for sample runs. The slope of this mean value of MSD as a function of time is used to compute the self-diffusivity. This is presented in Figure S4 and Figure S5 for 5 ns and 30 ns production runs respectively in the supplementary information.

The presented water model is developed using both TIP3P and SPC/E with parameters reported in the literature and compared with the values of self-diffusivity of water from these simulations to establish the transport properties. The values of diffusivity computed via our models namely, TIP3P and SPC/E are compared with these simulation results. The results for SPC/E water model are found to be in close agreement with experimental values found in literature. The self-diffusion coefficient of pure water has been experimentally measured to be 2.3 × 10^–9^ m^2^/s at 298 K using the diaphragm-cell technique or the pulsed-gradient spin-echo (PGSE) NMR method^31^. This validation of the water model, proven with experimental results ensures subsequent confidence in the results for pure water. Additionally, comparison with the Stokes–Einstein equation^32^ further validates our model. The proposed insilico model diffusivity results are verified with the tool SEGWE, which is a data-based model developed by researchers at Manchester University NMR Methodology Group^25^ as well as other recent available literature. The SEGWE tool offers improvisations and better prediction abilities using a combined analytical and data-driven approach complete with GUI^25^. Diffusivity values computed via SEGWE tool are presented in Figure S1 in the supplementary information.

Both water models TIP3P and SPC/E have been implemented and their effects assessed on the transport properties such as diffusivity to ascertain their utility as listed in Table 4. A rigorous comparison of the self-diffusivity values shows that the values computed using TIP3P is higher than what is seen in case of experimental data, and that given by the SEGWE tool by more than two times. This observation is in line with the viscosity values obtained earlier that had shown a low viscosity for water while using TIP3P model. Since, SPC/E model values are found to be in close agreement with both experimental and simulation results from literature, this model is implemented in our work.

**Table 4:** Simulated water self-diffusivity for pure water compared with experimental and SEGWE tool values.

Water Self diffusivity at 298 K—[10^–9^ m^2^/s]	
Insilico Model (water)—TIP3P model	5.64
Insilico Model (water)—SPC/E model	2.52
Experimental Values^33^	2.3
SEGWE^25^	2.128

### Comparison of glucose self diffusivity for an aqueous solution of glucose

As an initial step to understand the usability of SPC/E model of water as a base for insilico sweat model, we understand the transport characteristics of a biomarker such as glucose at a maximum composition present in sweat (see Table 1). Since glucose diffusivity values are available in literature both as simulations as well as experimental, it is selected as the candidate analyte. The values of glucose diffusivity are computed via molecular dynamics simulations and compared with the ones in the literature^34,35^. All the experiments and simulations were conducted at 25˚C for glucose in water system and the values thus experimentally arrived at were subsequently compared with the diffusivity values provided by SEGWE tool. The experimental studies presented were performed using a Spinco Model H diffusion apparatus as a Rayleigh interferometer^36^. Solvent–distilled and deionized water and sugar included as per molarity of 0.1 M. The values of diffusivity of glucose in water were reported as 0.63 × 10^–9^ m^2^/s at 298K^37^. The simulated value of glucose diffusivity at 298 K obtained in this work is 0.65 × 10^–9^ m^2^/s. However, comparing the values obtained using SEGWE tool we observe that it over-predicts more than what is seen in this simulation and in experiments. The slight variation from the Stokes–Einstein relationship^32^ is attributed to the non-spherical nature of the glucose molecule and the effects of micro viscosity around the molecules. Since glucose is nearly spherical, these effects can be attributed to microviscosity^34^.

Generally, mass transport can occur by three processes: migration, convection, and diffusion. Migration is the movement of ions in an electric field and does not occur for neutral molecules such as glucose, nor convection which is the bulk movement caused by external stimulus. Therefore, self-diffusion is the primary cause of the movement of the species and is considered for validation. As can be seen from the Table 5, the SPC/E water model is in close agreement with the modelling results and experimental data. The insilico model thus developed with water as the solvent with a proportionate number of glucose molecules is validated with literature data. The individual biomolecules are selected from protein data bank^18^ and added to the water molecules from the earlier SPC/E insilico model. As can be seen from the Table 5, the glucose diffusivity values given by the simulations at 310 K is 1.1 × 10^–9^ m^2^/s. The experimental literature shows that the self-diffusivity is slightly lower at 0.95 × 10^–9^ m^2^/s. These slight differences can be attributed to the small number of glucose molecules considered in the simulation as well as the slight errors introduced by considering the center of mass of glucose molecule for the various computations.

**Table 5:** Self diffusivity of glucose in an aqueous solution of glucose of a concentration of 200 µM at two temperatures (298 K and 313 K). The values from the present simulation are compared to the values from experiments and the computed values from the SEGWE model.

Glucose diffusivity at 298 K—[10^−9^ m^2^/s]		310 K—[10^−9^ m^2^/s]
Insilico Model (water + glucose)	0.63	1.1
Literature Values^35^	0.65	0.95
SEGWE^25^	0.681	0.930

### Comparison with sodium and chloride diffusivity for an aqueous solution of NaCl

The second step of the development involves validating the transport behaviour of the ions of Na^+^ and Cl^−^, a major component in sweat, in the aqueous solution. A simulation of NaCl of a concentration equal to 56 mM in water is simulated Na^+^ or Cl^−^ can be directly measured using ion-selective electrodes^6,7^ or electrical conductivity of the sweat can be measured, since Na^+^ and Cl^−^ are the abundant ions in sweat. These diffusion coefficients are available in the literature and can be compared with the simulated results to ensure the developed insilico model can be further developed into an insilico sweat model. These values are temperature-dependent, and a valid model will have significant utility provided all the diffusion coefficient values are in close agreement with the literature^38^. Table 6 shows the self-diffusivity of the Cl^-^ ions at 298 K. The simulated value of diffusivity is 1.55 × 10^–9^ m^2^/s. The experimental literature shows the value of 2.03 × 10^–9^ m^2^/s. SEGWE tool computes the diffusivity of to be equal to 1.489 × 10^–9^ m^2^/s. On the other hand, our simulated data and the experimental data of Cl^-^ diffusivity shows that it moves faster than the Na^+^ ion. However, the SEGWE tool predicts that Na^+^ ion moves faster than Cl^-^ ion. It indicates that MD simulation is able to capture the experimental behaviour of the NaCl aqueous solution.

**Table 6:** Self diffusivity of sodium and chloride in an aqueous solution of NaCl of a concentration of 56 mM at temperature 298 K. The values from the present simulation are compared to the values from experiments and the computed values from the SEGWE model.

Chloride diffusivity at 298 K—[10^–9^ m^2^/s]	
Insilico Model (water + NaCl)	1.36
Literature values^38^	2.03
SEGWE values^25^	1.489

Sodium diffusivity at 298 K—[10^–9^ m^2^/s]	
Insilico Model (water + NaCl)	1.45
Literature values^38^	1.6942
SEGWE values^25^	1.867

### Validation of insilico sweat model

In this section we describe the transport properties of an aqueous solution mixture consisting of NaCl (56 mM) and glucose in appropriate concentrations (200 µM) as that of eccrine sweat. The use of glucose as an analyte provides a way to observe the validity of the development of the model with the data available in the literature. This validation is the result of extensive simulations performed by the authors. Experimental values of the diffusivity and viscosity of aqueous solutions of salt and glucose from the literature are compared with the simulated values of insilico sweat model. Glucose is considered as a representative biomolecule for comparison of computed values of diffusivity and viscosity. Thus, validated this insilico model can be subsequently used for other candidate analyte biomolecules such as cortisol, uric acid, metabolites etc.

#### Validation at elevated temperatures and varying concentrations

Subsequently, the insilico sweat model was rigorously validated at elevated temperatures (310 K) and varying salt concentrations (10-100 mM) to simulate fever conditions and dehydration in subjects is presented in Table 7.

**Table 7:** Self diffusivity of species in aqueous solution of NaCl of a concentration of 56 mM at 310 K. The values from the insilico sweat model are compared to the values from experiments and the calculated values from the literature.

Comparison of viscosity and diffusivity values at 310 K		
	Insilico Sweat Model	Literature
Viscosity [mPa s]^37^	0.6942	0.6
Glucose Diffusivity [10^–9^ m^2^/s]^34^	1.1	0.93
Water [10^–9^ m^2^/s]	2.8	2.906

Comparison of the data with the values provided in literature for a salt concentration of 56 mM show good agreement. Similarly, the self-diffusivity of water also shows close agreement between the simulated values and the value provided in literature. At the same time, the diffusivity values of glucose are simulated to be 1.1 × 10^–9^ m^2^/s. The value in the literature is 0.93 × 10^–9^ m^2^/s for a glucose concentration of 66 mM. Encouraged by the validity of the insilico sweat model, we simulate the dehydration conditions of subjects by varying salt concentrations between 14 and 112 mM given that the range of compositions of salt found in eccrine sweat is between 10 and 100 mM. This simulates the transition from normal (14 mM) to dehydrated (112 mM) subjects. The variation in diffusivity of glucose shows that the values consistently decrease with increasing concentration of NaCl. However, the diffusivity values of water remain unchanged for the entire concentration range as listed in Table 8.

**Table 8:** Self diffusivity of species in aqueous solution of NaCl of a concentration of 14–112 mM at 298 K. The diffusivity values from the insilico sweat model are listed for water, sodium and glucose.

Comparison of diffusivity values of glucose and sodium with NaCl variation at 298 K					
NaCl conc. [M]	No of sodium/chloride ions	Water diffusivity [10^–9^ m^2^/s]	Sodium diffusivity [10^–9^ m^2^/s]	Chloride diffusivity [10^–9^ m^2^/s]	Glucose diffusivity [10^–9^ m^2^/s]
0.014	8	2.6	1.58	1.4	0.66
0.028	16	2.6	1.55	1.45	0.65
0.056	32	2.59	1.54	1.37	0.63
0.112	64	2.59	1.35	1.23	0.55

## Summary

The insilico eccrine sweat model is presented and validated as a tool to assist in the development of various wearable biosensors such as the ones to detect glucose, cortisol and other target analytes. This model will serve as an aid to multiple laboratory experiments and is offered as a stand-alone application complete with GUI for fellow researchers in this field. The applications for this work will serve as a horizontal means for a broad spectrum of users for design of bioreceptor element of wearable sensors for glucose, cortisol, metabolites, and other such analytes since experimentally conducting multiple such test conditions will require considerable time and the proposed insilico eccrine sweat model can augment these expensive experimental results. Such an insilico model for sweat is not present to the best of our knowledge and this work provides a building block in the direction of insilico model of eccrine sweat. This LAMMPS-based tool can simulate different eccrine sweat conditions such as temperature and concentration of NaCl and can further be automated to simulate circadian cycle. Thus, this validated insilico eccrine sweat model can serve as an aid to expedite the development de novo biosensors by addition of other analytes of interest e.g. cortisol, uric acid etc., simulate various temperatures and salt concentrations, expand search space for candidate target receptors by their binding affinity and assess the interference between species via simulations.

The slight variation observed in the values for diffusivity of glucose in water with various salt concentrations can be attributed to the micro-viscosity changes in the fluid. Additionally, the Stokes–Einstein equation assumes glucose to be a spherical molecule and neglects the micro-viscosity variations. The number of molecules of glucose is significantly less as compared to the salt ions and therefore the computed diffusivity may not have the averaging advantage. Besides the literature values available for the salt concentrations are slightly different from the simulated conditions. The available viscosity and diffusivity literature values via empirical equations and experiments for varying values of salt concentrations and temperatures nevertheless agrees well with the proposed insilico eccrine sweat model and therefore can be considered as a candidate tool for further research in this direction.

## Supplementary Information

Supplementary Information.

## Data Availability

The data and insilico sweat model tool developed that support this study are available upon reasonable request from the corresponding author.
