# Supplementary material for: Development of an insilico model of eccrine sweat using molecular modelling techniques
Source: Sci Rep. 2022 Nov 24;12:20263. doi: 10.1038/s41598-022-24440-x (PMC9691721; doi:10.1038/s41598-022-24440-x)
Supplement: Supplementary file 1 — Supplementary Information. [file 41598_2022_24440_MOESM1_ESM.pdf]

Supplementary Information

Development of an *insilico* model of eccrine sweat using molecular modelling techniques

Parijat Deshpande[1,2] Bharath Ravikumar[1] Siddharth Tallur[3] Debjani Paul[4] Beena Rai[1]

[1] TCS Research, Tata Research Development & Design Centre (TRDDC), Pune 411013, India
[2]Centre for Research in Nanotechnology & Science (CRNTS), IIT Bombay, Mumbai 400076, India.
[3]Department of Electrical Engineering, IIT Bombay, Mumbai 400076, India.
[4]Department of Biosciences and Bioengineering, IIT Bombay, Mumbai 400076, India.

E-mail: parijat.deshpande@tcs.com

This supplementary information provides representative plots for viscosity and diffusivity of the selected analytes within the *insilico* model. Namely, Glucose, Sodium and Chloride and base solvent water. The proposed model is developed incrementally as per the flow diagram below and simulated values of diffusivity and viscosity at various concentrations (10-100mM) of NaCl and temperatures (298K and 310K) are validated with literature data. These ranges are selected to depict human subjects with dehydrated and fever conditions. Glucose in included as a candidate biomolecule in the concentration 200 µM and is representative of various analytes of interest present in trace quantities. The values of viscosity and diffusivity are then validated to arrive at an *insilico* eccrine sweat model intended to aid the development of a biosensing element of the final wearable biosensor.

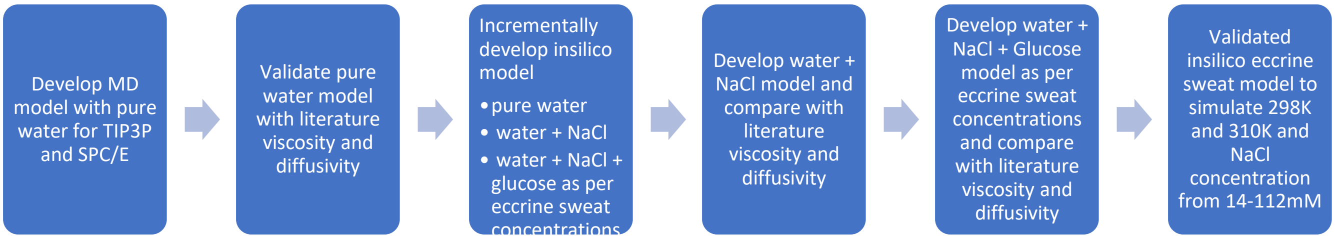

Initially, both TIP3P and SPC/E water models are considered, and the corresponding values of diffusivities of sodium, chloride and glucose are compared with literature to arrive at the final *insilico* model using SPC/E water model. This *insilico* model is further developed by replicating the 35Å cube 8 times with periodic boundary conditions to ensure the results are comparable with the macro-properties of the biofluid (eccrine sweat).

The plots include computed values via LAMMPS commands as listed below:

# Diffusivity of water

```
compute     msd1 water msd com yes
fix         9 water vector 10 c_msd1[4]
variable    Waterdiff equal slope(f_9)/6/(10*dt)
fix         wdiff all ave/time 100 1 100 v_Waterdiff file Water_Diff.txt
```

The proposed *insilico* model diffusivity results are verified with the tool SEGWE, which is a data-based model developed by researchers at Manchester University NMR Methodology Group[25] as well as other recent available literature.

Table 1: Computed values of diffusivity for various analytes using SEGWE Tool

Analytes @ Temperatures	Diffusivity × 10^{-9} m^2 /s
Glucose in water at 298K	0.681
Glucose in water at 310K	0.93
Na in water at 298K	1.86
Na in water at 310K	2.44
Cl in water at 298K	1.48
Cl in water at 310K	2.03
Water in water 298K	2.128
Water in water 310K	2.906

Figure S1 : SEGWE Computations

Glucose in water at 298K – **SEGWE value 0.681 [10^{-9} m^2 /s]**

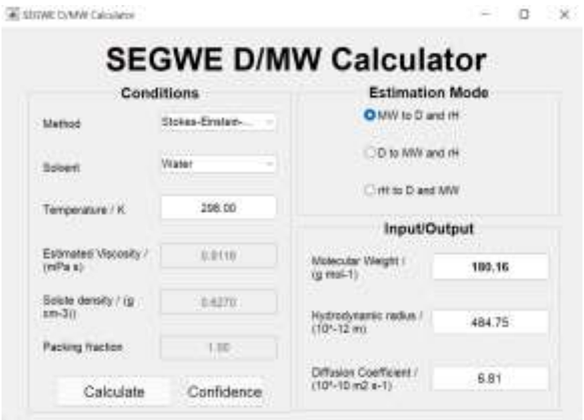

Glucose in water at 310K **SEGWE value 0.930 [10^{-9} m^2 /s]**

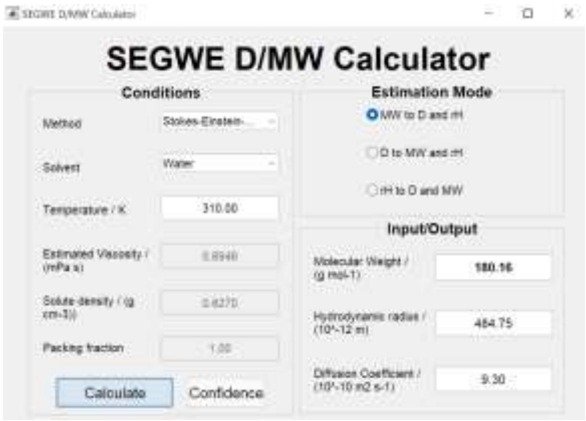

Na in water at 298K **SEGWE value 1.86 [10^{-9} m^2 /s]**

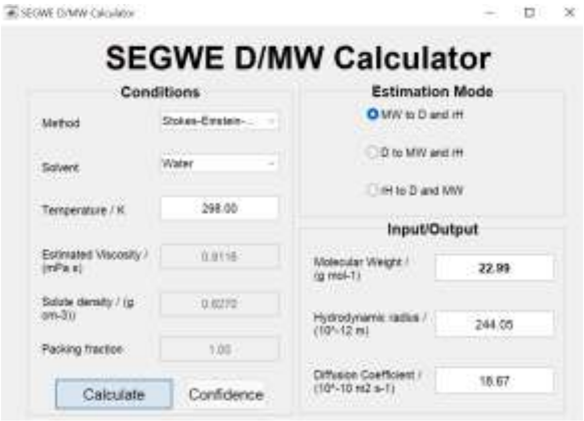

Na in water at 310K **SEGWE value 2.44 [10^{-9} m^2 /s]**

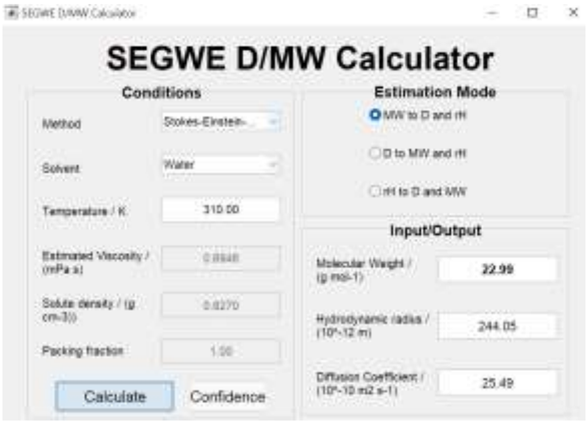

Cl in water at 298K **SEGWE value 1.48 [10^{-9} m^2 /s]**

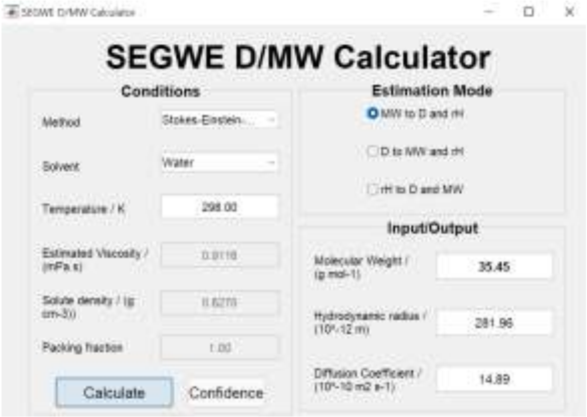

Cl in water at 310K **SEGWE value 2.03 [10^{-9} m^2 /s]**

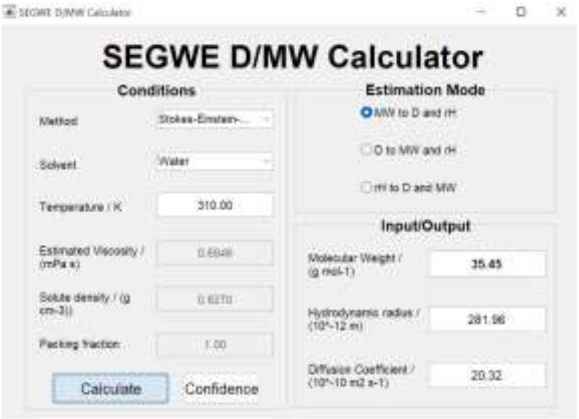

Water in water 298K **SEGWE value 2.128 [10^{-9} m^2 /s]**

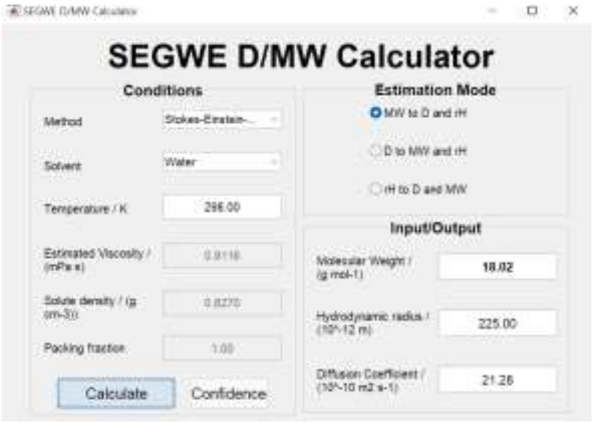

Water in water 310K **SEGWE value 2.906 [10^{-9} m^2 /s]**

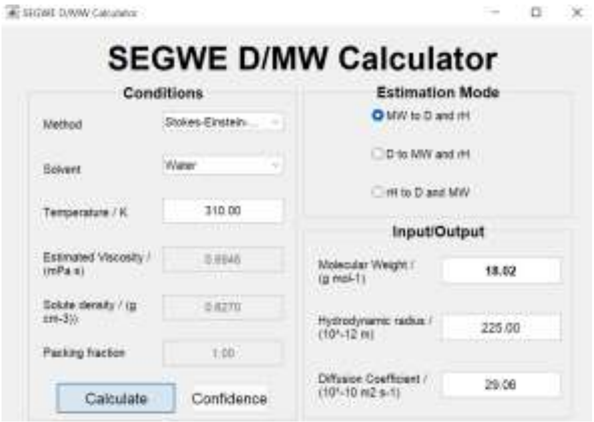

Representative plots of viscosity and diffusivities of Water, Sodium, Chloride and Glucose are provided in this document as results of numerous simulations and serve as a validation of the *insilico* sweat model.

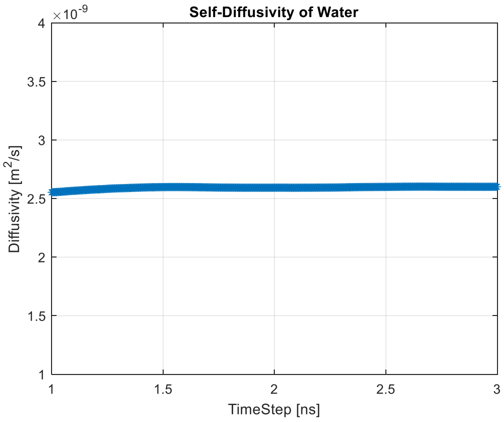

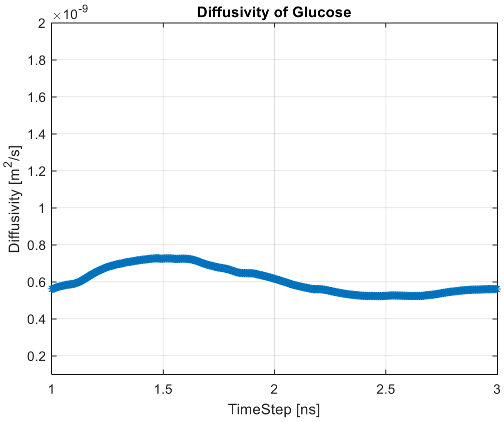

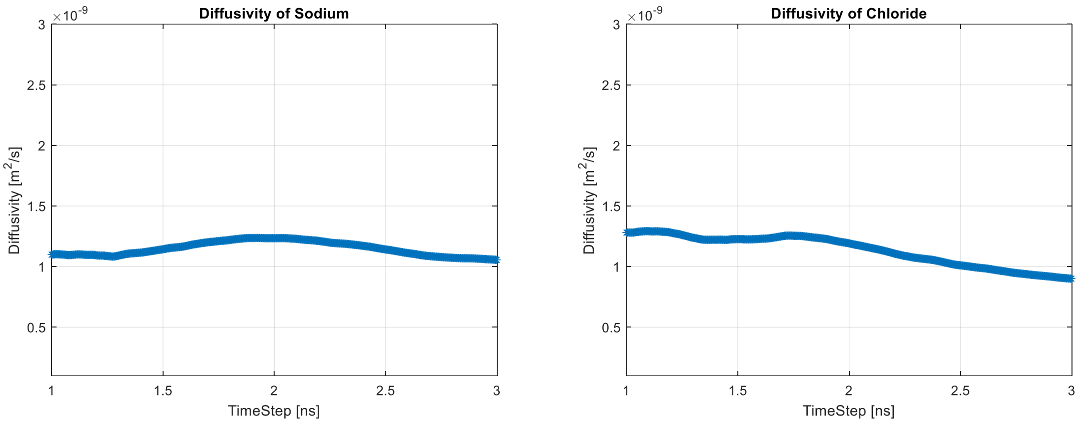

Figure S2: Representative simulations for an aqueous solution of NaCl (56mM) and Glucose (200 µM) with SPC/E water model at 298 K

30 ns Production Run Water + Salt (NaCl conc. 56mM) + Glucose 200µM at 298K

The results are plotted below along with the MSD plots as per the reviewer's instructions for extended simulation time. The points show the simulated data and the line shows the linear fit to the MSD that leads to a self-diffusivity values in close agreement with the diffusivity plots. It can be seen from the plots that the values of diffusivity stabilize within reasonable range.

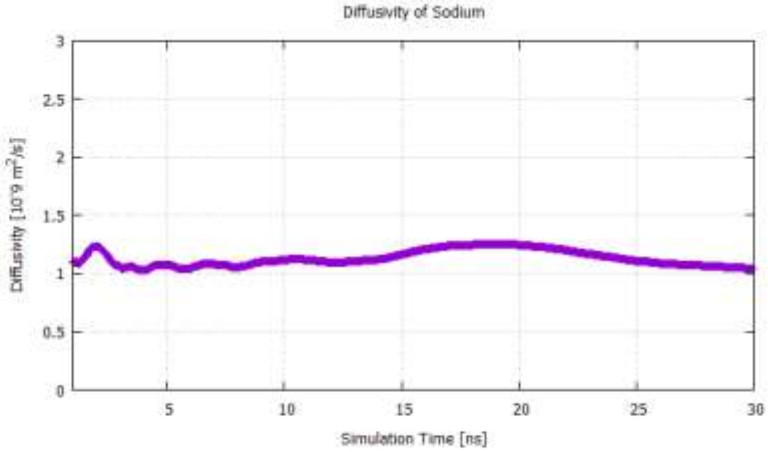

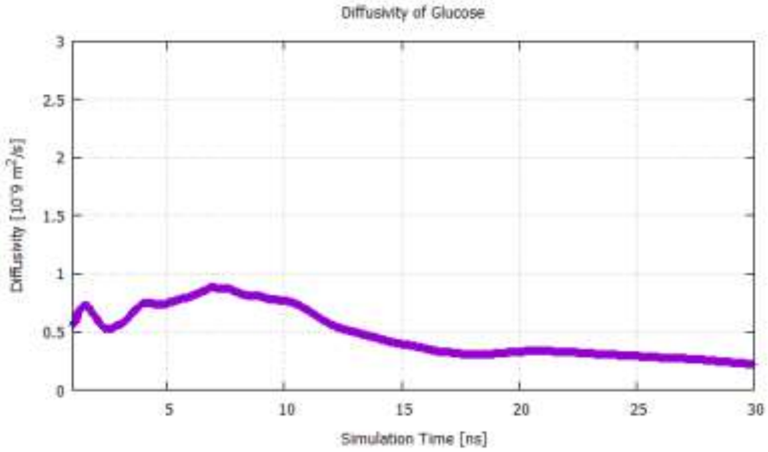

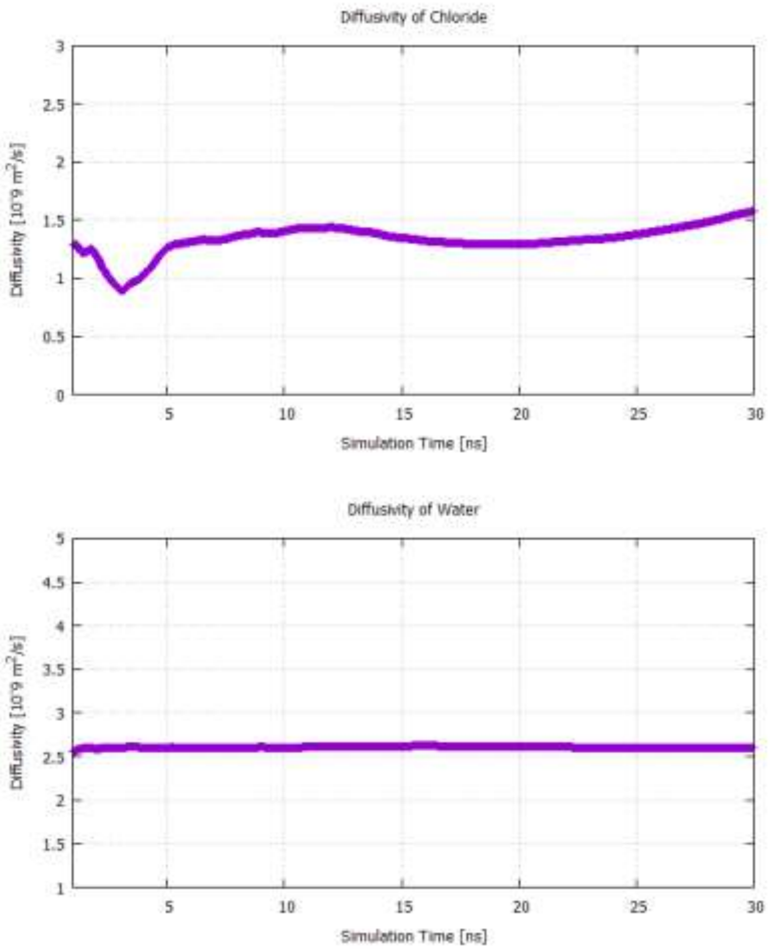

Figure S3: Representative simulations for an aqueous solution of NaCl (56mM) and Glucose (200 µM) with SPC/E water model at 298 K for 30ns

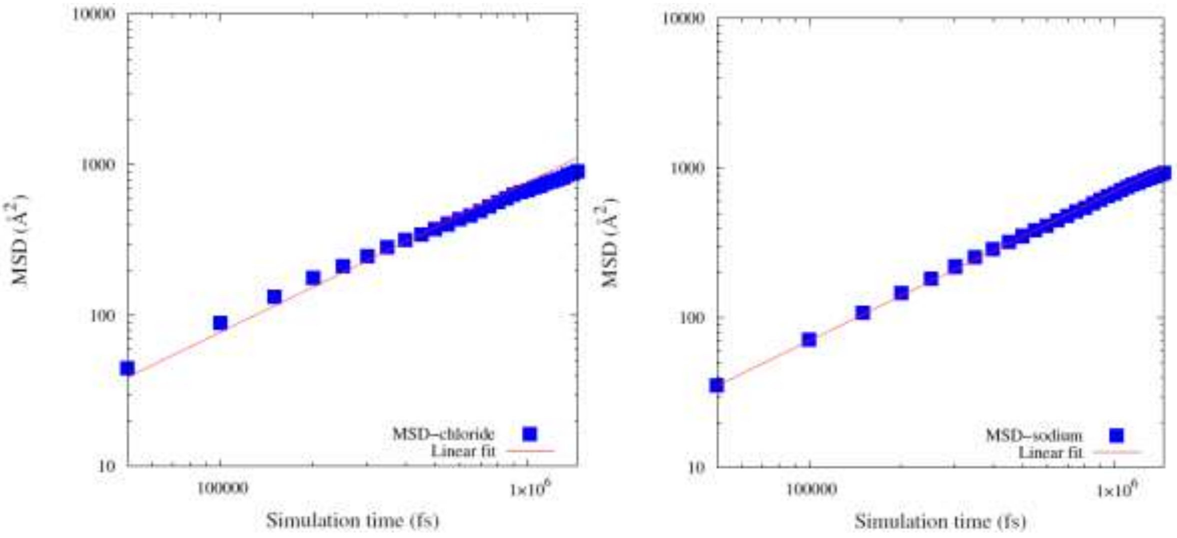

Figure S4: MSD of sodium and chloride ions as a function of time computed using multiple time origins at 298 K for an NaCl concentration of 56mM in the insilico sweat model for a single run.

The points show the simulated data, and the line shows the linear fit. The slope gives the corresponding diffusivity values of sodium 1.55 × 10^{-9} m^2 /s and chloride 1.4 × 10^{-9} m^2 /s.

Similarly **for longer duration simulation runs of 30 ns**, the fit is linear as depicted in the plots below for Chloride and Sodium.

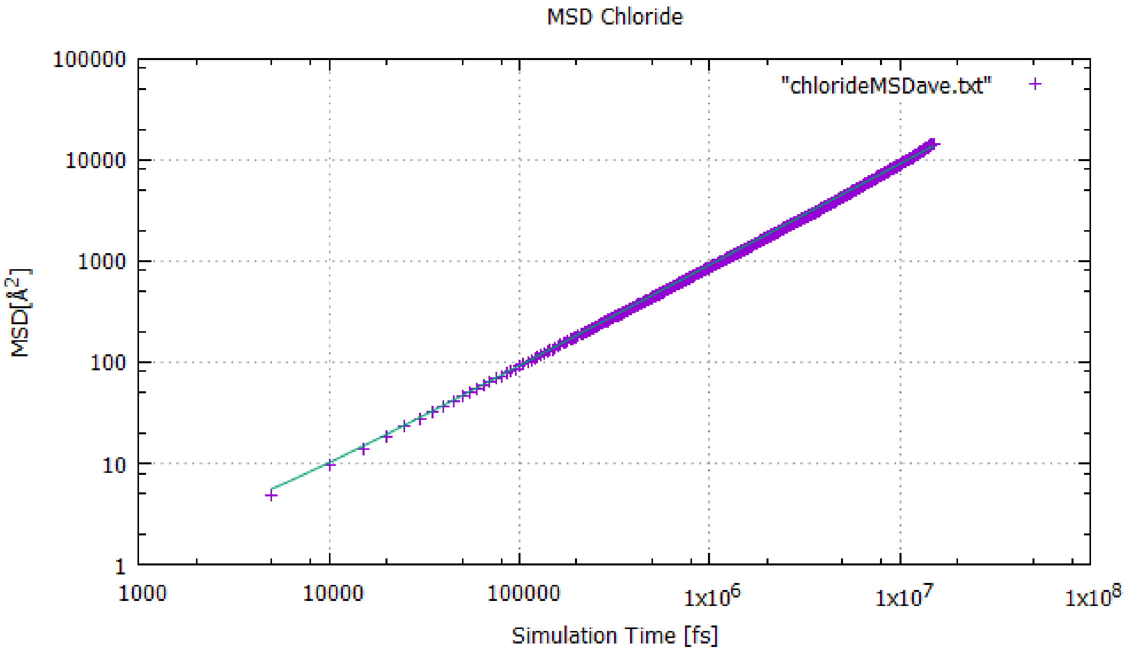

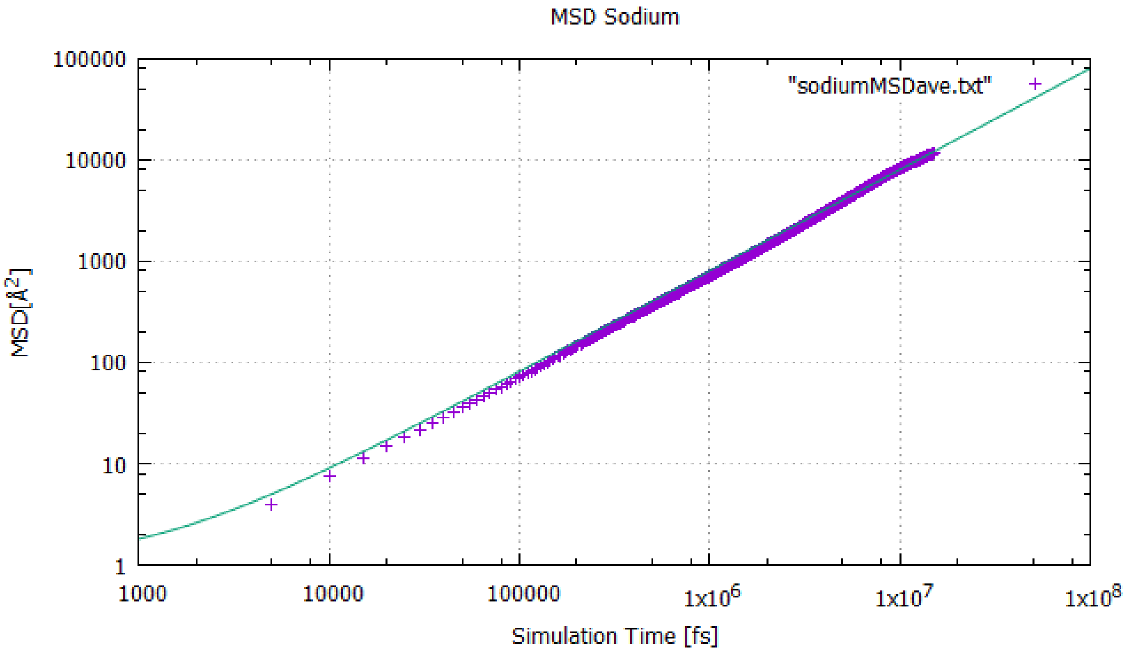

Figure S5: MSD of sodium and chloride ions as a function of time computed using multiple time origins at 298 K for an NaCl concentration of 56mM in the insilico sweat model for a single run.

The points show the simulated data, and the line shows the linear fit. The slope gives the corresponding diffusivity values of sodium 1.54 × 10^{-9} m^2 /s and chloride 1.37 × 10^{-9} m^2 /s.

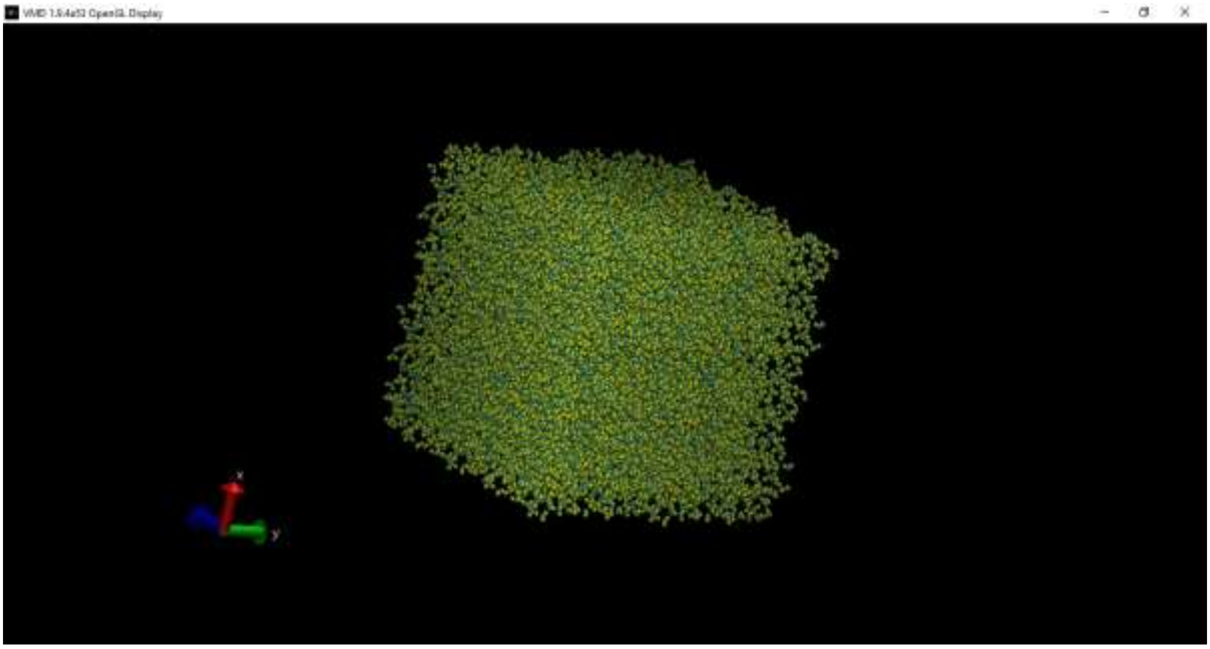

Figure S6: A VMD rendering of the *insilico* model of the simulation system containing water molecules, sodium and chloride ions and glucose molecules at 298 K for an NaCl concentration of 56mM in the energy minimized condition.
